# Physical activity and health-related quality of life among physiotherapists: a cross sectional survey in an Australian hospital and health service

**DOI:** 10.1186/1745-6673-9-1

**Published:** 2014-01-09

**Authors:** Steven M McPhail, Monique C Waite

**Affiliations:** 1 Queensland University of Technology, School of Public Health and Social Work and Institute of Health and Biomedical Innovation, Victoria Park Road, Kelvin Grove 4059, Queensland, Australia; 2 Centre for Functioning and Health Research, Princess Alexandra Hospital, Metro South Health, Corner of Ipswich Road and Cornwall Street, Buranda 4102, Queensland, Australia

**Keywords:** Physical activity, Work related musculoskeletal disorders, Physiotherapists, Prevention, Injury, Attrition, Quality of life, Workforce, Australian

## Abstract

**Background:**

Physiotherapists are a professional group with a high rate of attrition and at high risk of musculoskeletal disorders. The purpose of this investigation was to examine the physical activity levels and health-related quality of life of physiotherapists working in metropolitan clinical settings in an Australian hospital and health service. It was hypothesized that practicing physiotherapists would report excellent health-related quality of life and would already be physically active. Such a finding would add weight to a claim that general physical activity conditioning strategies may not be useful for preventing musculoskeletal disorders among active healthy physiotherapists, but rather, future investigations should focus on the development and evaluation of role specific conditioning strategies.

**Methods:**

A questionnaire was completed by 44 physiotherapists from three inpatient units and three ambulatory clinics (63.7% response rate). Physical activity levels were reported using the Active Australia Survey. Health-related quality of life was examined using the EQ-5D instrument. Physical activity and EQ-5D data were examined using conventional descriptive statistics; with domain responses for the EQ-5D presented in a frequency histogram.

**Results:**

The majority of physiotherapists in this sample were younger than 30 years of age (n = 25, 56.8%) consistent with the presence of a high attrition rate. Almost all respondents exceeded minimum recommended physical activity guidelines (n = 40, 90.9%). Overall the respondents engaged in more vigorous physical activity (median = 180 minutes) and walking (median = 135 minutes) than moderate exercise (median = 35 minutes) each week. Thirty-seven (84.1%) participants reported no pain or discomfort impacting their health-related quality of life, with most (n = 35,79.5%) being in full health.

**Conclusions:**

Physical-conditioning based interventions for the prevention of musculoskeletal disorders among practicing physiotherapists may be better targeted to role or task specific conditioning rather than general physical conditioning among this physically active population. It is plausible that an inherent attrition of physiotherapists may occur among those not as active or healthy as therapists who cope with the physical demands of clinical practice. Extrapolation of findings from this study may be limited due to the sample characteristics. However, this investigation addressed the study objectives and has provided a foundation for larger scale longitudinal investigations in this field.

## Background

Physiotherapists (also known as physical therapists in some regions) are a professional group with a high rate of attrition [1-4]. Many physiotherapists leave the profession, or at least cease clinical practice, relatively early in their career [1-3]. Wolpert and Roshida reported that raising a family, as well as seeking new challenges and further education as important contributors to physiotherapist attrition [1]. Additionally, Scutter and Goold [5] Martinussen and colleagues [6], and Pavlakis and colleagues [7] reported the burnout phenomenon as another important contributor to the attrition of physiotherapists. However, a range of investigations have indicated work related musculoskeletal disorders also pose a substantial threat to the career longevity and health-related quality of life of physiotherapists working in clinical practice [8-13].

Work related musculoskeletal disorders have been documented across a range of professions and clinical settings within the healthcare industry [8,9,11,14-22]. Physiotherapists have been identified as one professional group with particularly high risk of work related musculoskeletal disorders [11,17,20,22]. It has previously been reported by Cromie, Robertson and Best that 80% of physiotherapists may experience symptoms of work related musculoskeletal disorders in at least one body region over a one-year timeframe [11]. These authors also reported that over the course of their career, as many as 91% will experience a work related musculoskeletal disorder [11]. The rate of recurrence has been reported to be as high as 88% [22,23].

The personal and economic impacts of these conditions are profound. Approximately one in six physiotherapists has reported that a work related musculoskeletal disorder has prompted them to leave the profession or change their area of specialty [11]. Work-related musculoskeletal conditions also carry economic costs to individuals and healthcare organizations that employ physiotherapists through sick leave entitlements, healthcare intervention costs and loss of productivity [11,24]. These personal and organizational impacts have prompted efforts to prevent work-related musculoskeletal disorders among physiotherapists through the publication of health and safety briefings, as well as identification of potential risk factors and prevention strategies [11,19,20,25].

A salient, and potentially unavoidable, risk factor for musculoskeletal disorders among physiotherapists is the physically demanding nature of routine tasks completed during their clinical practice [23]. Prior studies in this field have consistently identified the physical demands of clinical practice as a contributor to musculoskeletal conditions among therapists [17,26-28]. Passier and McPhail reported that some of the demands of clinical practice that have been identified by physiotherapists as potential risk factors for musculoskeletal disorders included: being required to work in the same posture for long periods; bending or twisting; reaching and working away from the body; carrying, lifting or moving heavy materials or equipment; lifting or transferring dependent patients; unanticipated sudden patient movements or falls; working with bariatric patients; performing the same tasks repeatedly; and treating many patients in one day [23].

As experts in the musculoskeletal system, prevention and treatment of musculoskeletal disorders and the clinical practice requirements of their profession, physiotherapists have themselves offered insight into potential strategies for the prevention of musculoskeletal disorders related to their work [23]. Potential prevention strategies have been previously grouped into six broad categories: physical conditioning, organizational strategies, workload and work allocation, work practices, environment and equipment, and education and training [23]. The efficacy of strategies that may fall into these categories remains largely unknown, and a multi-factorial strategy may prove to be the most effective [23]. However, to date there have been limited investigations to help determine which types of interventions are (and are not) worthy of further development and evaluation.

This study has two inter-related primary objectives. The first is concerned with one of the aforementioned potential categories for work related musculoskeletal disorder prevention strategies; physical conditioning. The physical condition of physiotherapists is likely to be influenced by their lifestyle, health state and occupational behaviors. In this way, physiotherapists themselves may be able to improve or maintain their physical conditioning without (or with) the support of the organizations in which they work. This is in contrast to some of the other potential strategies for reducing work-related musculoskeletal disorders that are almost entirely dependent on organizational structures and supports [23]. The second objective of the present study is related to the health-related quality of life of physiotherapists in a broader sense. With the exception of being at risk of musculoskeletal disorders, not much is known about the health-related quality of life of practicing clinical physiotherapists.

Therefore this preliminary investigation aimed to examine the self-reported physical activity levels and health-related quality of life of a sample of physiotherapists working in a metropolitan region of Australia.

It was hypothesized that despite being at high risk of work-related musculoskeletal disorders, practicing physiotherapists would report high levels of health-related quality of life; perhaps with the exception of some reports of pain or discomfort (potentially from musculoskeletal disorders). It was also hypothesized that most practicing physiotherapists would already be meeting minimum recommended guidelines of at least 150 minutes of moderate intensity physical activity across 5 sessions per week [29] either due to the nature of their work or a propensity of individuals who intrinsically value exercise to be working in this profession.

## Methods

### Study design

A cross sectional survey was undertaken.

### Participants and setting

Practicing clinical physiotherapists from three participating hospital based ambulatory clinics, and three subacute inpatient rehabilitation units were invited to participate in this investigation (n = 69). These clinics and rehabilitation units were all part of an Australian metropolitan hospital and health service. The inpatient rehabilitation units included geriatric rehabilitation (treating older patients with a range of primary diagnoses), brain injury rehabilitation, and spinal cord injury rehabilitation. The ambulatory clinics included an Orthopedic Physiotherapy Specialist Clinic and Multidisciplinary Disciplinary Service, an Aquatic Physiotherapy Clinic (also known as hydrotherapy) and a general physiotherapy outpatient clinic (treating patients with a range of primary diagnoses). Within this health service, clinical physiotherapists may work in more than one clinical area simultaneously or consecutively over time; including both inpatient units and ambulatory clinics. However, physiotherapists were not permitted to complete the questionnaire more than once. There were no other exclusion criteria. This variety of clinical settings was selected in order to ensure representation across a range of common metropolitan clinical contexts where physiotherapists practice.

This study received ethical approval from the Human Research Ethics Committees of the Metropolitan South Hospital and Health Service, and the Queensland University of Technology. Participation was voluntary and physiotherapists provided informed consent prior to taking part in this investigation.

## Materials

The authors considered it very important to minimize the burden associated with participating in this investigation in order to maximize the participation rate and questionnaire completion rate among the small available pool of potential participants comprising of busy clinical practitioners. Therefore, a concise questionnaire that addressed the research aims was considered the most appropriate for this investigation ahead of more burdensome methodologies, such as direct monitoring of physical activity or an expansive health questionnaire battery. The survey contained questions related to biographic information including gender, age, and years in the physiotherapy profession, as well as self-reported physical activity using the Active Australia survey [30] and health-related quality of life measured using the EQ-5D questionnaire [31].

The Active Australia Survey includes a self-report of physical activity completed in the previous week. Participants are required to detail the number of weekly occurrences and total duration of the following types of activity: continuous walking for at least 10 minutes; vigorous gardening or heavy work around the yard; vigorous physical activity (for example, jogging or cycling); and other moderate physical activities (for example, swimming or social tennis). The number and duration of activities reported are not limited to formal cardiovascular exercise, but may include any type of physical activity. Physical activity reported on this instrument is classified into walking, moderate or vigorous activity including duration and number of activities in each of the categories [30]. For the purpose of summating total moderate intensity physical activity time equivalent, the time spent undertaking vigorous activities is assigned a double weighting. For example, 20 minutes of high intensity sporting activity would be assigned a 40 minute equivalent value toward the summative total of moderate physical activity. Sufficient physical activity is calculated as the accumulation of at least 5 sessions and 150 minutes of moderate physical activity (or equivalent vigorous activity) per week [30].

This instrument was developed and validated as part of an Australian government initiative to assist in the collection of uniform, standardised data for physical activity measurement among Australian adults [30]. An expert working group on physical activity measurement was established by the Australian Institute of Health and Welfare to develop the questionnaire content. This group reviewed existing physical activity measures, examined the issues surrounding measurement of physical activity, undertook related research and consulted widely before identifying data elements necessary for physical activity measurement [30]. This expert group developed the Active Australia Survey as a means to collect data consistent with the aforementioned data elements after drawing on content from a range of previously used questionnaires. The Active Australia Survey has been used in a variety of nation-wide and state-based government surveys in Australia and has exhibited good reliability, face validity, criterion validity and respondent acceptability [30].

The EQ-5D is a generic health-related quality of life questionnaire developed by the Euroqol group, an international network of multidisciplinary researchers [31]. This instrument includes the domains of mobility, personal care, usual activities, pain / discomfort and anxiety / depression; for which respondents indicate that they either experience no problems, some/moderate problems or extreme problems. The instrument also includes a 101 point vertical visual analogue scale where 0 (0 cm) and 100 (20 cm) are represented by the worst and best imaginable health state respectively [31]. Responses from the five domains can be converted to a single summary score (known as multi-attribute utility) where death and full health are represented by 0.00 and 1.00 respectively [32,33]. The EQ-5D is among the most widely used instruments internationally for the evaluation of health-related quality of life [34-40] and has demonstrated favourable reliability [34-37], validity [35-40] and responsiveness [35,37,40,41] across a range of populations [34-41].

### Procedure

Physiotherapists were invited to participate in the study during a routine staff meeting. At this time, physiotherapists were given opportunity to take a paper copy of the questionnaire for completion at a time convenient to them. Survey return post boxes were placed in each of the clinic or rehabilitation unit staff rooms, and a research assistant attended three consecutive weekly staff meetings to collect completed surveys.

### Analysis

**Figure 1:**
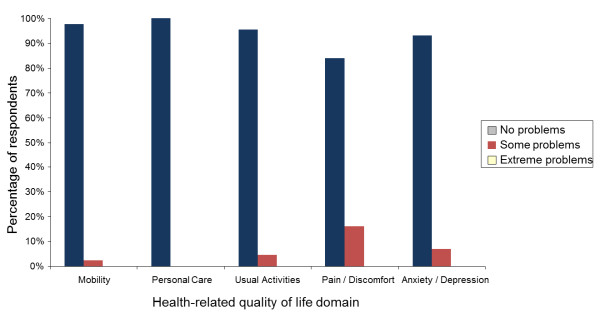
Health-related quality of life across the five domains of the EQ-5D instrument.

**Figure 2:**
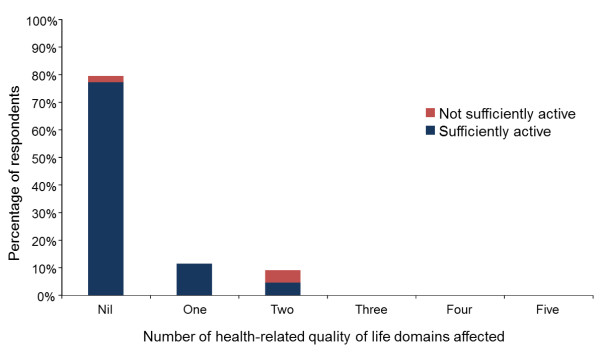
Total number of affected health-related quality of life domains reported by each individual when completing the EQ-5D instrument.

**Figure 3:**
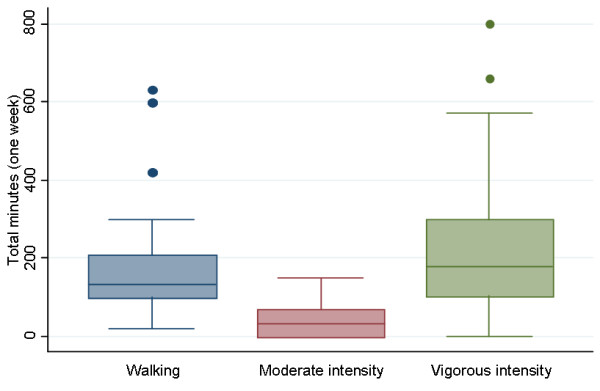
Box plots of physical activity duration reported by physiotherapists for walking, moderate, and vigorous physical activity in the previous week.

Demographic information was analyzed descriptively (median and inter-quartile range (IQR), number and percentage) and presented in Table 1. Median and interquartile range were considered more appropriate for describing non-normally distributed data than mean and standard deviations (SD). Multi-attribute utility scores were derived from the EQ-5D responses using the Dolan tariff system [33]. This method was used as it was derived from a society with similar cultural values to the sample and has demonstrated favorable psychometric properties in previous investigations [34-39,41]. Results from the five domains of the EQ-5D instrument were presented in a histogram representing the frequency (expressed as a percentage of respondents) of self-reported problems across each domain (Figure 1). Additionally, the number of domains that respondents reported problems in is displayed in Figure 2. The number and proportion of participants whose physical activity levels were sufficient to be of benefit to their health was determined according to previously reported guidelines [30]. Physical activity levels measured from the Active Australia Survey questions were presented in box plots (Figure 3). Physical activity levels were also analyzed descriptively (median and IQR) and tabulated for respondents who did and did not meet the minimum recommended physical activity guidelines (Table 2). Stata/IC (StataCorp, Version 11.2) was used for data analysis.

**Table 1:** Characteristics of physiotherapist participants

	**Total respondents**
	**n = 44**
Age	
20–29 years (%)	25 (56.8%)
30–39 years (%)	15 (34.1%)
≥ 40 years (%)	4 (9.1%)
Female (%)	30 (68.2%)
Median (IQR) years working in the profession	3 (1–9)
Health-related quality of life	
Median (IQR) utility score*	1.00 (1.00-1.00)
Median (IQR) EQ-VAS	80 (70–90)

* The mean (standard deviation) Australian Population Norm utility score is 0.83 (0.20), where 0.00 represents death and 1.00 represents full health [45].

**Table 2:** Median (inter-quartile range) time in minutes per physical activity intensity and number of sessions reported

	**Walking**	**Moderate**	**Vigorous**	**Total**	**Physical activity**
	**time**	**activity**	**activity**	**activity**	**sessions**
Sedentary	-	-	-	-	-
n = 0 (0%)					
Insufficient	80.0	0.0	0.0	87.5	5.0
n = 4 (9.1%)	(57.5-101.3)	(0.0-0.0)	(0.0-3.8)	(68.8-101.3)	(4.5-5.3)
Sufficient*	150.0	50.0	210.0	450.0	16.0
n = 40 (90.9%)	(120.0-217.5)	(0.0-80.0)	(120.0-300.0)	(315.0-665.0)	(12.0-19.0)

* National Physical Activity Guidelines of at least 150 minutes of moderate physical activity per week, across at least 5 sessions of physical activity [29] calculated according to Active Australia Survey procedures [30].

## Results

The survey was completed by 44 physiotherapists; equating to a response rate of 63.7%. Each of these 44 data sets had responses for all questions (100% completion rate). Demographic information for the participants is presented in Table 1. The majority of respondents were female (n = 30, 68.2%), consistent with the gender distribution of physiotherapists in these clinical settings [3,22,23]. Respondents’ ages ranged from 20 to 51 years (median age = 28.5, IQR 25–34.3 years), and their years of experience working as a physiotherapist ranged from less than 1 year to 30 years. The majority (n = 25, 56.8%) were younger than 30 years of age.

Health-related quality of life data are displayed in Table 1 (summary multi-attribute utility and health state visual analogue scale data) and Figure 1 (health-related quality of life domains). The EQ-5D multi-attribute utility score ranged from 0.73 to 1.00 with the majority of participants (n = 35, 79.5%) being in full health (as derived from the EQ-5D); consistent with the first study hypothesis. The overall health state visual analogue scale scores ranged from 69 to 100. In each of the five EQ-5D domains, the majority of physiotherapists reported no problems (Figure 1). The most frequently reported problems were in the pain or discomfort domain (n = 7, 15.9%), followed by anxiety and depression (n = 3, 6.8%), usual activities (n = 2, 4.6%), and mobility (n = 1, 2.3%). No participants reported extreme problems for any domain.

The number of effected domains reported by participants is displayed in Figure 2. Pain and discomfort was the most frequently reported problem occurring in isolation (n = 4), but also co-occurred with anxiety and depression (n = 2) and difficulty with usual activities (n = 1). Among physiotherapists classified as being insufficiently physically active, only one reported no problems in any health-related quality of life domain; the other insufficiently active respondents reported that two domains of their health-related quality of life were affected.

According to their self-reported physical activity levels (Table 2), the majority of participants (n = 40, 90.9%) met the minimum requirements for sufficient activity for health and no participants were sedentary; consistent with the second study hypothesis. Box plots presenting physical activity time by intensity are displayed in Figure 3. Overall the respondents engaged in more vigorous physical activity (median time = 180 minutes) and walking (median time = 135 minutes) than for moderate exercise (median time = 35 minutes) each week.

## Discussion

### Main finding

This investigation indicated that the majority of physiotherapists in the sample were already physically active and in good health-states; confirming the study hypotheses. Not only had physiotherapists frequently undertaken walking activities that may be associated with their employment, but also vigorous physical activities (e.g. jogging).

This finding adds weight to a claim that general physical activity conditioning strategies [42-44] may not be useful for preventing musculoskeletal disorders (among active healthy physiotherapists), but rather, future investigations should focus on the development and evaluation of role specific conditioning strategies.

Physiotherapists in this study were also relatively young with the majority under 30 years of age; consistent with prior investigations of physiotherapists working in similar clinical settings [1,22,23,26]. Given the younger age range, and physical activity levels reported, it was not surprising that the overall health profile of physiotherapists in this sample was also very good. Physiotherapists reported considerably better health-related quality of life than the broader Australian population [45]. A large proportion of physiotherapists in this sample did not report deficits in any of the EQ-5D domains. Pain or discomfort was the most commonly affected health-related quality of life domain among physiotherapists who did report some problems, but this was not a very high proportion of the sample (15.7%).

### Interpretation

There are at least four possible reasons for relatively infrequent reports of health-related quality of life being impacted by pain or discomfort among this professional group at high risk of work related musculoskeletal disorders. First, it is possible that previous studies have over-reported the risk of work related musculoskeletal disorders among physiotherapists. This seems unlikely, given the consistency between reports from a range of previous studies in this field [11,17,20,22,23]. Second, the sample was relatively young, with less than 10% of the sample over 40 years of age. It is plausible that the likelihood of having a work related musculoskeletal disorder that causes some pain or discomfort may increase with age and time spent working in physically demanding roles. However, it is noteworthy that the majority and perhaps most serious work related musculoskeletal disorders occur during the first five years of physiotherapists’ clinical practice [11,22,23]. A third (and meritorious) potential reason for relatively infrequent reports of pain or discomfort, is that physiotherapists who had experienced a substantial work related musculoskeletal disorder may no longer be working in these clinical settings having already left the profession or moved to a less physically demanding role and would therefore not have been represented in the sample [11,46]. Finally, it is noteworthy that physiotherapists are skilled in managing musculoskeletal disorders, perhaps including those which they have sustained. Those who were currently working in these clinical settings may not have any pain or discomfort impacting their health-related quality of life at the time of questionnaire completion because they had already recovered from any previously sustained musculoskeletal conditions [23].

There are several caveats that should be taken into account when considering the high physical activity levels reported by physiotherapists in this sample. This study sample was adequate to address the research aims at hand for the target region; however, survivorship bias (also known as the healthy worker effect in occupational studies [46]) is an important consideration that limits the ability to extrapolate the findings of this study beyond comparable clinical samples. Survivorship bias may occur through an inherent attrition of physiotherapists that are not as fit or healthy as those therapists who cope with the physical demands of clinical practice. Attrition of this nature may contribute to a predominantly young, fit and healthy workforce being present in any cross-section of physiotherapists from clinical settings; similar to the samples observed in this and other studies [1,2,22,23,26]. However, it is also noteworthy that it would be unfair to attribute the high rates of physiotherapist attrition from clinical practice solely to musculoskeletal disorders. A range of other stressors and motivators for leaving clinical practice beyond the scope of this study have been reported, perhaps the most notable of which is the burnout phenomenon [6,47,48].

### Implication for strategies to prevent work related musculoskeletal disorders

The main implication from this investigation is that general physical activity conditioning interventions successfully implemented in other occupational environments are unlikely to be useful for physiotherapists currently working in these clinical settings. While general physical activity conditioning may be useful for some less physically active physiotherapists, the majority of physiotherapists working in clinical practice among this sample were already very active. On the other hand, the development and evaluation of a role specific conditioning intervention is worthy of consideration. Additionally, non-physical conditioning related strategies (including organization based strategies) may prove to be more effective in prevention of musculoskeletal disorders among healthy active physiotherapists working in clinical settings [23].

### Strengths and limitations

This research investigation has several strengths and limitations. The satisfactory response rate and absence of missing data may be considered strengths of this investigation owing to the use of concise survey instruments and persistent follow-up for survey collection. In a concerted effort to minimise questionnaire completion burden and maximise response rate, the investigators decided against the inclusion of a suite of questions examining the nature of specific musculoskeletal conditions, and instead utilised questionnaire content directly addressing the research aims for this study. The authors considered it particularly important to maximize the response rate given the relatively small number of physiotherapists available for recruitment among the participating clinics and inpatient units from this region. Additionally, previous investigations have already reported the nature and recurrence rates of musculoskeletal disorders among physiotherapists. While this sample was adequate to address the research aims among physiotherapists in this region, physiotherapists from dissimilar societal contexts may not have responded in the same way.

Another possible limitation in using a self-reported health-related quality of life questionnaire was that a reference bias, recall bias or response shift may also have occurred among physiotherapists working in this setting [49-52]. In other words, physiotherapists in these clinical settings frequently work with patients in very poor health states. It is plausible that unless the physiotherapists were currently experiencing substantial health-related quality of life impairment, they may not have considered it appropriate to report anything other than being in a very good health state. However, the authors considered that this was not an excessive risk given that physiotherapists have both recalled and openly reported work related musculoskeletal disorders in previous investigations [8-11,18,23,24].

It is also noteworthy that self-reported physical activity may overestimate or underestimate actual physical activity performed [53]. In the context of the very high physical activity levels reported by the majority of physiotherapists working in these clinical settings, even if a moderate overestimation or underestimation had occurred, this would not have affected the overarching conclusion that clinical physiotherapists from these settings were already frequently undertaking both low intensity walking as well as vigorous physical activities.

## Conclusions

Physical-conditioning based interventions for the prevention of musculoskeletal disorders among practicing physiotherapists may be better targeted to role or task specific conditioning rather than general physical conditioning among this physically active population. It is plausible that an inherent attrition of physiotherapists may occur among those not as active or healthy as therapists who cope with the physical demands of clinical practice. Extrapolation of findings from this study may be limited due to the sample characteristics. However, this investigation addressed the study objectives and has provided a foundation for larger scale longitudinal investigations in this field. Future research may also consider physical activity and health-related quality of life among physiotherapists in non-clinical practice roles or specifically target experienced physiotherapists. Further research in other regions with dissimilar social contexts to metropolitan Australia may also prove valuable to this field of research.

## Competing interests

The authors declare no conflict of interest.

## Authors' contributions

SMM contributed to study conception, design, coordination, analysis, and principal drafting of the manuscript as well as appraisal and editing. MCW contributed to analysis and drafting the manuscript as well as appraisal and editing. Both authors read and approved the final manuscript.
